# Hip Fracture Registry and Audit Readiness in Southeast Asia: A Scoping Review and Public-Evidence Implementation Gap Map

**DOI:** 10.7759/cureus.117520

**Published:** 2026-10-06

**Authors:** Guan Hao Chester Tan, Nicholas T Yew

**Affiliations:** 1 Department of Orthopaedic Surgery, Changi General Hospital, Singapore, SGP; 2 Department of Orthopaedic Surgery, Singapore Armed Forces Medical Corps, Singapore, SGP

**Keywords:** clinical audit, fracture liaison service, hip fracture, orthogeriatrics, registry, southeast asia

## Abstract

Hip-fracture registries and audit systems support feedback, benchmarking, and quality improvement, but the public visibility of this infrastructure across Southeast Asia is unclear. This review mapped publicly identifiable registry readiness, audit, care quality, and secondary prevention evidence for hip-fracture care across Association of Southeast Asian Nations (ASEAN) countries.

We conducted a scoping review and public-evidence implementation gap map across the 10 ASEAN member states. Searches covered PubMed/MEDLINE, Embase, and Cumulative Index to Nursing and Allied Health Literature (CINAHL) and were supplemented by grey-literature searches. Included sources were evaluated against registry readiness and care quality audit domains, and country-domain evidence was scored from publicly traceable evidence.

Of 1,294 records identified, 810 unique records were screened, 122 sources were sought, 111 were assessed and 59 were included in the final evaluation. Evidence was strongest in Singapore, Malaysia, the Philippines, and Thailand, but consisted of different infrastructure types: historical national registry evidence, multicentre databases, institutional registries, linked administrative data, hospital audits, and fracture liaison service datasets. No eligible, publicly accessible recurring annual hip-fracture audit report or benchmarking output was identified for any included country.

Publicly identifiable hip-fracture registry and audit evidence in Southeast Asia is emerging but uneven. A staged regional minimum dataset and transparent audit framework could help convert institutional experience into sustainable national and regional quality-improvement systems.

## Introduction and background

Hip fracture is a high-impact fragility fracture associated with mortality, loss of independence, institutionalisation, and substantial health-system cost. As populations age across Southeast Asia, hip-fracture care is likely to become an increasing orthopaedic, geriatric, rehabilitation, and public-health burden. Improving outcomes requires more than operative capacity alone. Timely surgery, orthogeriatric co-management, delirium prevention, early mobilisation, falls assessment, osteoporosis assessment and treatment, secondary fracture prevention, follow-up, and outcome surveillance all influence recovery after hip fracture.

Hip-fracture registries and audit systems provide a practical mechanism for making these care processes visible. Established audit programmes can define minimum datasets, measure care standards, provide feedback to hospitals, benchmark performance, and create a platform for quality improvement. International and regional initiatives have increasingly emphasised the need for hip-fracture datasets, dashboards, and implementation support, particularly in low- and middle-income settings where routine outcome measurement may be limited [1-5].

Their clinical value lies in linking routinely collected data to measurable care standards, feedback, and corrective action. By making variation in surgery timing, orthogeriatric assessment, mobilisation, bone-health treatment, and outcomes visible, a registry or audit can support improvement across the patient pathway rather than merely describe activity.

Despite this momentum, Southeast Asia has no clearly visible regional hip-fracture registry infrastructure. Published and public sources suggest that some countries have institutional registries, national orthopaedic registry components, or fracture liaison service databases, whereas others have guideline or osteoporosis care signals without clear registry or audit evidence. For clinicians, societies, and policymakers planning hip-fracture audit implementation, it is important to distinguish mature registry infrastructure from local audits, planned initiatives, and broader secondary-prevention context.

This study aimed to map publicly identifiable hip-fracture registry readiness, audit, care-quality, and secondary-prevention evidence across Southeast Asia. We developed a country-domain implementation gap map to distinguish mature registry/audit infrastructure, institutional or planned systems, and contextual public evidence; absence of eligible public evidence was not interpreted as absence of service activity.

## Review

Methods

We conducted a scoping review and implementation gap map of hip-fracture registry readiness and related fragility-fracture care quality audit domains in Southeast Asia. The review included all 10 Association of Southeast Asian Nations (ASEAN) member states: Brunei Darussalam, Cambodia, Indonesia, Laos, Malaysia, Myanmar, the Philippines, Singapore, Thailand, and Vietnam. The purpose was to map publicly traceable evidence rather than to rank health systems or outcomes.

For this review, registry readiness was defined as publicly traceable evidence of capacity to identify a hip-fracture population, collect standardised patient-level data, measure one or more care processes or outcomes, and support governance, feedback, or reporting. A formal registry was an organised, ongoing system with defined data collection and governance; a local audit was a time-limited or service-level assessment against standards; an institutional database was a hospital- or programme-based dataset without evidence of broader audit functions; and routine clinical documentation alone was not treated as registry or audit infrastructure. Registry readiness was used as a descriptive mapping construct and was not an externally validated index.

Database searches were conducted in PubMed/MEDLINE, Embase, and Cumulative Index to Nursing and Allied Health Literature (CINAHL). Search terms combined hip fracture or fragility fracture terms with registry, audit, database, quality improvement, fracture liaison service, orthogeriatric care, osteoporosis, secondary fracture prevention, and the ASEAN countries.

A structured grey-literature search was also conducted to identify publicly available evidence across official government, professional-society, registry/audit, hospital-programme, International Osteoporosis Foundation, Capture the Fracture, Fragility Fracture Network, and Asia Pacific Fragility Fracture Alliance sources. Search terms included ASEAN countries with hip-fracture registry, hip-fracture audit, fragility fracture, fracture liaison service, osteoporosis guideline, orthogeriatric care, time to surgery, and secondary fracture prevention terms.

Eligible sources were peer-reviewed articles, reports, protocols, registry or audit descriptions, retrospective database studies, guidelines, and official institutional or service webpages that were publicly accessible or locally archived and contained extractable country-level evidence for at least one prespecified domain. Institutional reports and webpages were included only after direct review of the underlying document or page. Retrospective databases were included when their population, setting, data source, and relevant care-process or outcome information were stated. Sources limited to general epidemiology, unrelated orthopaedic services, elective arthroplasty registries, or non-specific routine documentation without implementation relevance were excluded. Planned initiatives, working groups, and organisational statements could contribute limited readiness evidence but were not classified as functioning registry or audit systems.

Two reviewers independently screened titles and abstracts, assessed retrieved full texts or public-source records against the prespecified eligibility criteria, and independently charted eligible evidence. Disagreements regarding eligibility, source role, domain assignment, or score were resolved through discussion and consensus; no third-party adjudication was required.

The search comprised 1,294 raw records or observations: 579 database records and 715 grey-literature sources. After removal of duplicates, 810 were screened. A total of 122 records were sought; 11 could not be retrieved. A total of 111 records were assessed for eligibility, and 59 public-evidence sources were ultimately included in the evidence synthesis. The study selection process is shown in Figure 1.

**Figure 1:**
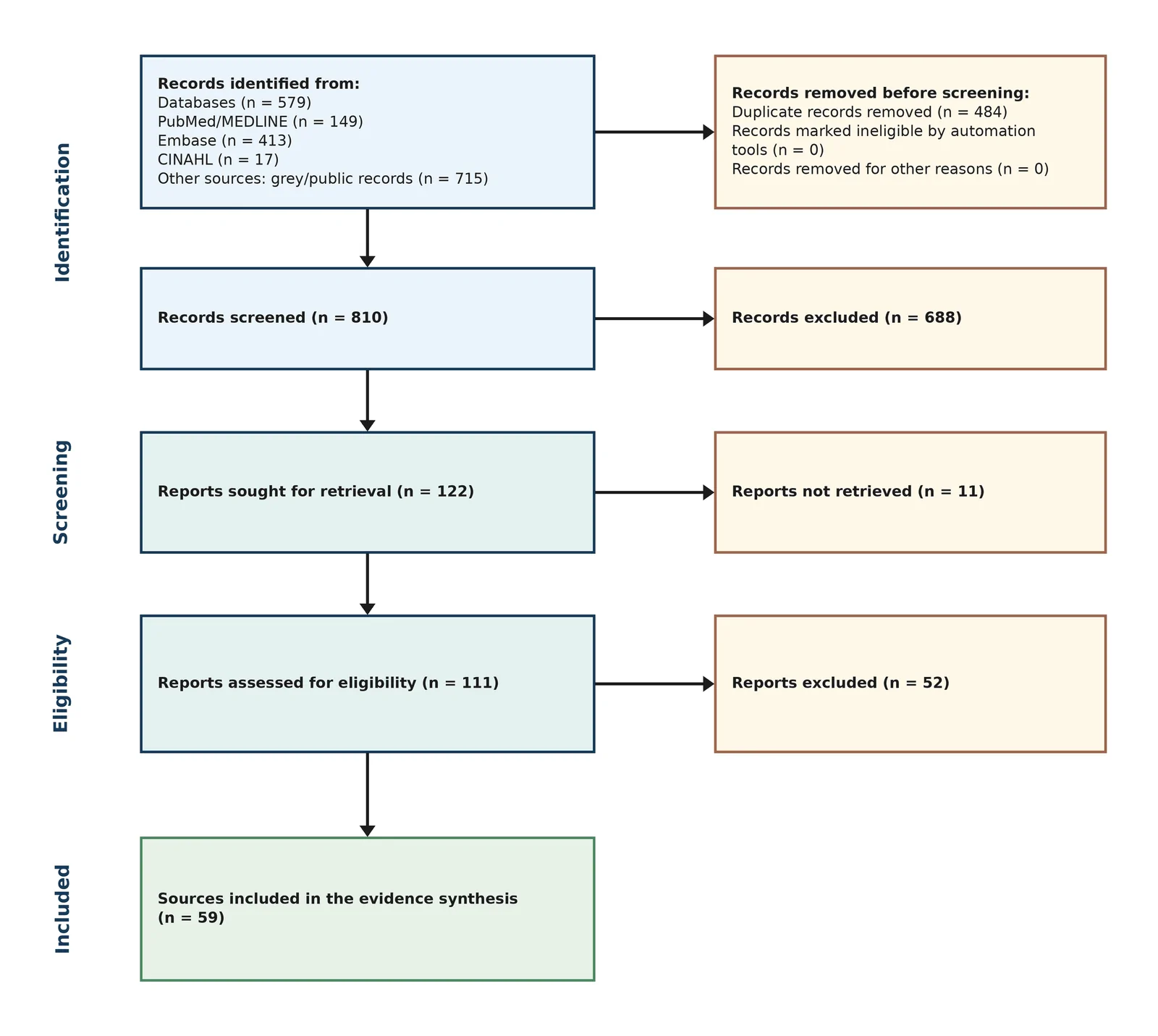
PRISMA 2020 flow diagram.

Information from 14 domains was extracted: registry or audit system; national guideline or pathway; time-to-surgery target or reporting; orthogeriatric co-management; delirium or cognitive assessment; early mobilisation or rehabilitation; falls assessment or prevention; osteoporosis assessment; anti-osteoporosis medication initiation; fracture liaison service; mortality reporting; functional or discharge outcome reporting; public annual reporting or benchmarking; and funding, governance, or sustainability.

Country-specific domain scores were assigned by consensus from the strongest eligible publicly traceable evidence using a prespecified four-level guide: 0, no eligible public evidence identified; 1, limited, local, planned or contextual evidence; 2, functioning institutional, multicentre, or repeated programme evidence without established national coverage; and 3, direct national or public-system evidence. Scores describe public visibility and implementation level, not health-system performance; a score of 0 does not establish absence of a service. Duplicate and overlapping records were reconciled by title, digital object identifier (DOI)/PubMed identifier (PMID), URL, organisation, and year. When multiple sources described the same programme, they were linked rather than treated as independent evidence of separate infrastructure. When evidence differed in scope, maturity, or reporting level, the final judgment reflected the strongest directly relevant eligible source, while the source trace recorded the supporting evidence and interpretation limit. The scoring rubric was developed for this mapping exercise and is not externally validated.

The 14 domains were anchored to elements of mature hip-fracture audit systems, including minimum common dataset work, national registry comparison studies, and publicly reported audit programmes such as the National Hip Fracture Database (NHFD) in England, Wales, and Northern Ireland and the Australian and New Zealand Hip Fracture Registry (ANZHFR) [2,4,6-11]. The comparisons illustrate how standardised data capture, clinical process indicators, outcome measures, feedback, and public reporting can be combined to support quality improvement. The domains were used to assess publicly visible progression towards these audit functions.

This review was not prospectively registered because database and structured grey-literature searches had commenced before registration was considered.

Results

Included Evidence and Regional Pattern

A total of 59 sources met the eligibility criteria for registry readiness, audit, database, surveillance, care-quality, or benchmarking evidence. Source types included multicentre database studies, national registry studies, institutional registry cohorts, administrative database-linkage studies, quality improvement audits, protocols, implementation toolboxes, guidelines, programme pages, scoping reviews, and conference abstracts. The evidence was concentrated in Malaysia, the Philippines, Singapore, and Thailand. The country-domain public-evidence map is presented in Table 1. The complete 59-source inventory is provided in the Supplementary Table included in the Appendices.

**Table 1:** Country-domain public-evidence map for hip-fracture registry readiness in Southeast Asia. Lao PDR: Lao People's Democratic Republic; AOM: anti-osteoporosis medication; FLS: fracture liaison service.

Country	Registry/ audit	Guideline/pathway	Surgery timing	Orthogeriatrics	Delirium/cognition	Mobilisation/rehab	Falls	Osteoporosis assessment	AOM initiation	FLS	Mortality	Function/discharge	Public benchmarking	Governance	Total/42
Brunei Darussalam	2	1	1	2	0	0	2	1	1	1	0	2	0	1	14
Cambodia	0	0	0	0	0	0	0	0	0	0	0	0	0	0	0
Indonesia	0	1	1	0	0	1	1	1	1	0	0	0	0	0	6
Lao PDR	0	0	0	0	0	0	0	0	0	0	0	0	0	0	0
Malaysia	2	1	2	1	2	2	2	2	2	2	1	0	0	1	20
Myanmar	1	1	0	0	0	1	1	1	1	2	0	0	0	1	9
Philippines	2	1	2	2	2	2	1	1	1	2	2	2	0	1	21
Singapore	3	1	1	2	0	2	2	2	2	2	3	2	0	1	23
Thailand	1	1	3	2	2	2	2	2	2	2	2	2	0	0	23
Vietnam	0	1	1	0	0	0	0	1	1	2	0	0	0	1	7

Country-Specific Synthesis

Brunei: Sources identified a single-hospital hip-fracture admission audit with care process and functional data, demonstrating some institutional audit visibility. A hip-fracture pathway proposal and an orthogeriatric liaison service were early signals of early implementation [12-14].

Cambodia and Laos: No eligible country-specific public source was retrieved. This sparse public-evidence profile is limited by public availability, indexing, and language constraints.

Indonesia: Indonesia had updated osteoporosis guidance involving Perhimpunan Osteoporosis Indonesia (PEROSI), the Ministry of Health, and the Indonesian Rheumatology Association, alongside publicly reported hip-fracture surgery waiting times of more than three days, fracture-risk assessment, treatment criteria linked to reimbursement policy, and falls-prevention exercise recommendations. Fragility Fracture Network Indonesia also identified rehabilitation as a collaboration focus. Together, these sources demonstrate early public activity across guideline, care process, secondary prevention, and rehabilitation domains [15,16].

Malaysia: Malaysia had historical orthopaedic registry experience, including hip-fracture registry implementation in public hospitals and reported variation in time to surgery. Public evidence also described early postoperative mobilisation, falls-prevention activity, osteoporosis assessment and treatment initiation, fracture liaison service provision, including 28 services listed on the Capture the Fracture Map of Best Practice, and a hospital hip-fracture model of care incorporating multidisciplinary and postoperative care standards. These sources demonstrate historical registry infrastructure alongside contemporary institutional care quality and secondary prevention activity [1,3,17-25].

Myanmar: Myanmar showed limited registry readiness evidence alongside contextual osteoporosis, falls, rehabilitation, and secondary prevention signals [26-28].

Philippines: The Philippines had multicentre online fragility hip-fracture database evidence across 12 hospitals using a modified minimum common dataset, with additional orthogeriatric/fracture liaison service database activity. This demonstrates multicentre data capture and several care process/outcome signals [2,7,29-36].

Singapore: Singapore had strong institutional hip-fracture registries, administrative or claims data linked to mortality information, perioperative quality improvement data, functional follow-up, and secondary fracture-prevention infrastructure. These sources demonstrate substantial institutional and linked-data capacity [9,37-46].

Thailand: Thailand had hospital and fracture liaison service registries/databases, fast-track programme monitoring, mortality and functional-outcome reporting, and a planned surveillance/prevention programme. The 2025 International Osteoporosis Foundation (IOF) country audit reports an annually reported fast-track hip-surgery-within-72-hours indicator and a new surveillance/prevention system with data collection beginning in July 2025; this country-level annual indicator is not equivalent to a recurring comparative hip-fracture audit or public benchmarking programme [3,47-56].

Vietnam: Evidence identified a Ministry of Health osteoporosis guideline, an average hip-surgery waiting-time category (>3 days), and five publicly listed fracture liaison services [57,58].

Domain Synthesis

Registry, database, and audit infrastructure: The country profiles show heterogeneous levels and forms of audit readiness. Malaysia has historical orthopaedic registry evidence alongside contemporary institutional activity; the Philippines has experience using a minimum common dataset across multiple centres; Singapore has institutional registries and linked administrative data; and Thailand has hospital and fracture liaison service databases, with fast-track monitoring reported in selected programmes. These are important forms of data capture, but they are not interchangeable with a current, recurring national audit and public benchmarking [1-3,7,20-26].

Care-process and outcome visibility: Across Singapore, Thailand, the Philippines, Malaysia, and Brunei, one or more sources reported mortality, functional outcomes or discharge disposition, surgery timing, rehabilitation, or secondary-prevention activity. However, source types, denominators, and reporting cycles differed substantially. Some data and audit-related activities are publicly visible, but the available reporting is not sufficiently standardised to support valid comparisons of care performance [2,13,14,22,34-37,39,41-46,49-56,59].

Secondary prevention, public reporting, and governance: Fracture liaison service (FLS) and osteoporosis care activity was visible in several countries, but evidence of a national hip-fracture audit, national case ascertainment, or public comparative reporting was not identified. Although isolated data and some audit-related activities were publicly visible, none met the prespecified criterion for a recurring, publicly accessible national hip-fracture audit or benchmarking report. All 10 countries received a score of 0 for public annual reporting and benchmarking within the searched and included evidence set. This finding should not be interpreted as proof that services, internal audits, or non-public reporting do not exist.

Comparator Systems: What Mature Audit Adds

The UK NHFD and ANZHFR are functional mature systems that function as points of comparison. Their distinguishing features are standardised datasets, repeated data collection across participating sites, routine clinical indicators, feedback tools, annual public reporting, and mechanisms for identifying variation. The NHFD 2025 annual report describes report-level key performance indicators, survival analysis, and hospital-level outlier monitoring, including delirium assessment, mobilisation after surgery, and bone-health actions [10]. The ANZHFR evaluates performance against seven Hip Fracture Clinical Care Standard statements, including presentation, pain, orthogeriatric care, timing of surgery, mobilisation or weight bearing, prevention of another fracture, and transition from hospital care, and publishes the results in a Clinical Care Standard report with a supplementary e-report [11].

Discussion

This scoping review and public-evidence implementation gap map identifies distributed regional capability rather than a uniformly mature regional hip-fracture audit system. Evidence was concentrated in Malaysia, the Philippines, Singapore, and Thailand, but the infrastructure differed: historical registry evidence, multicentre databases, institutional registries, linked administrative data, hospital audits, and fracture liaison service datasets. Brunei adds directly extractable single-centre audit evidence, whereas Indonesia, Myanmar, and Vietnam demonstrate more contextual or institutional signals. Cambodia and Laos had no eligible country-specific evidence retrievable.

The domain synthesis clarifies the implementation gap. Patient-level data collection and selected outcome reporting are already visible, but the source base is uneven and does not yet demonstrate consistent common definitions, recurrent cross-site feedback, complete case ascertainment, or public comparative reporting.

We propose a staged implementation pathway: common case definitions and a feasible minimum dataset in sentinel hospitals; governance, data-quality procedures, and routine site feedback; followed by wider coverage and proportionate public reporting when completeness and stakeholder oversight permit responsible comparison [4,5,60,61]. Next steps could include evaluation of the pathway’s feasibility, cost, stakeholder acceptance, implementation burden, or sustainability.

NHFD and ANZHFR illustrate the additional functions that transform local data capture into mature audit: standardised datasets, repeated collection, clinically meaningful indicators, feedback cycles, and transparent reporting. These systems are useful functional references, but the staged pathway described here is a proposal rather than an implementation recommendation tested by this review. A sentinel-hospital approach may allow countries with existing registry or fracture liaison service experience to contribute early while enabling settings with limited public evidence to begin with low-burden core fields and explicit case definitions [10,11].

The study has material limitations. It maps publicly identifiable evidence and may miss internal, non-English, non-indexed, or inaccessible systems. Grey/public sources vary in stability and authority. Scores are conservative, source-dependent, and not psychometrically validated; they must not be interpreted as health-system performance measures. Within-country variation is likely to be substantial: evidence from a well-developed hospital, registry, or fracture liaison service should not be interpreted as evidence that equivalent infrastructure, access, or performance is present at other institutions in the same country. Validation with national orthopaedic and geriatric societies, fracture liaison service networks, ministries, and registry custodians is necessary before policy prioritisation can begin.

## Conclusions

Publicly traceable hip-fracture registry, audit, and care quality evidence across Southeast Asia is heterogeneous. The region has meaningful local and multicentre foundations, but the included evidence does not demonstrate mature, recurring public national hip-fracture audit and benchmarking. A staged common dataset, governance structure, and feedback cycle could connect existing registries, hospital audits, and fracture liaison services into sustainable national and regional quality improvement systems. Public comparative reporting should follow when case ascertainment, data completeness, and stakeholder oversight are sufficiently mature to make it responsible.

## Appendices

**Table 2:** Public-evidence source inventory. LMICs: low- and middle-income countries.

No.	Full Vancouver citation	Country/jurisdiction	Setting/level	Source type	Evidence contribution	Scope/interpretation	Source route
1	Cheah MH, Lai PSM, Ong T [1]. Implementation of a fracture liaison service in a tertiary hospital in Malaysia: a feasibility study. Arch Osteoporos. 2024, 19:70. 10.1007/s11657-024-01427-x. Available from: https://doi.org/10.1007/s11657-024-01427-x.	Malaysia	Institutional	Journal article	Final country-domain evidence: Malaysia—Falls assessment or prevention, osteoporosis assessment, anti-osteoporosis medication initiation, fracture liaison service.	Institutional service-level evidence.	Database
2	Tabu et al. [2]: Improving fragility hip fracture care through data: a multicentre experience from a country with an emerging economy during the COVID-19 pandemic. BMJ Open Qual. 2023, 12:e002299. 10.1136/bmjoq-2023-002299. Available from: https://doi.org/10.1136/bmjoq-2023-002299.	Philippines	Multicentre	Journal article	Final country-domain evidence: Philippines—Registry or audit system, time-to-surgery target or reporting, orthogeriatric co-management, delirium or cognitive assessment, early mobilisation or rehabilitation, falls assessment or prevention, mortality reporting, functional or discharge outcome reporting, funding, governance, or sustainability.	Multicentre implementation evidence.	Database
3	Tabu et al. [3]: Service availability and readiness for hip fracture care in low- and middle-income countries in South and Southeast Asia. Bone Jt Open. 2023, 4:676-681. 10.1302/2633-1462.49.bjo-2023-0075.r1. Available from: https://doi.org/10.1302/2633-1462.49.bjo-2023-0075.r1.	Malaysia; Thailand	Multicountry	Journal article	Final country-domain evidence: Malaysia—Time-to-surgery target or reporting, delirium or cognitive assessment, early mobilisation or rehabilitation, falls assessment or prevention, osteoporosis assessment; Thailand—Early mobilisation or rehabilitation.	Multicountry comparative evidence.	Database
4	Johansen et al. [4]: Developing a minimum common dataset for hip fracture audit to help countries set up national audits that can support international comparisons. Bone Joint J. 2022, 104-B:721-728. 10.1302/0301-620x.104b6.bjj-2022-0080.r1. Available from: https://doi.org/10.1302/0301-620x.104b6.bjj-2022-0080.r1.	International/comparator	National	Journal article	Contributes a minimum-dataset framework for registry/audit and public-benchmarking domains.	National policy and implementation evidence.	Database
5	Asia Pacific Fragility Fracture Alliance; Fragility Fracture Network [5]. APFFA-FFN Hip fracture registry toolbox: a resource to support registry implementation. (2021). Accessed: May 18, 2026: https://apfracturealliance.org/hfr-toolbox/	International/toolbox	Country/institutional context	Registry implementation toolkit/guidance	Contributes a registry-implementation framework for registry/audit and governance/sustainability domains.	Institutional implementation evidence.	Database
6	Klappenbach R, Lartigue B, Beauchamp M, Boietti B, Bosque L, Monteverde E [6]. P165: Hip fracture registries in low and middle income countries: a scoping review. Geriatr Orthop Surg Rehabil. 2021, 12:21514593211058764. 10.1177/21514593211058764. Available from: https://doi.org/10.1177/21514593211058764.	Regional/multicountry (LMICs)	Regional	Conference proceedings abstract	Corroborates registry/audit and governance/sustainability domains.	Regional implementation evidence.	Database
7	Tabu IA, Sandoval MAS, Mangubat AAS, Ching Bing- Agsaoay DD, Rosanna Pena MA [7]. Fragility hip fractures in a team-based multidisciplinary orthogeriatric care setting with a fracture liaison service: patients’ profile and early outcomes. Osteoporos Sarcopenia. 2019, 5:S3. 10.1016/j.afos.2019.12.006. Available from: https://doi.org/10.1016/j.afos.2019.12.006.	Philippines	Multicountry	Conference abstract/journal supplement	Final country-domain evidence: Philippines—Falls assessment or prevention, osteoporosis assessment, anti-osteoporosis medication initiation.	Multicountry comparative evidence.	Database
8	Khor et al. [8]: 65 fragility fracture network (FFN) Malaysia - a call to action for better outcome in Malaysia. Age Ageing. 2019, 48:iv13-iv17. 10.1093/ageing/afz164.65. Available from: https://doi.org/10.1093/ageing/afz164.65.	Malaysia	Country/institutional context	Conference abstract	Corroborates fracture-liaison-service and governance/sustainability domains.	Institutional implementation evidence.	Database
9	Chandran M, Tan MZ, Cheen M, Tan SB, Leong M, Lau TC [9]. Secondary prevention of osteoporotic fractures--an "OPTIMAL" model of care from Singapore. Osteoporos Int. 2013, 24:2809-17. 10.1007/s00198-013-2368-8. Available from: https://doi.org/10.1007/s00198-013-2368-8.	Singapore	Country/institutional context	Journal article	Final country-domain evidence: Singapore—Falls assessment or prevention, osteoporosis assessment, anti-osteoporosis medication initiation.	Institutional implementation evidence.	Database
10	International Osteoporosis Foundation [12]. Brunei Darussalam country report. (2025). Accessed: May 19, 2026: https://www.osteoporosis.foundation/sites/iofbonehealth/files/2025-11/Brunei_Final.pdf	Brunei Darussalam	Country level	Country report	Final country-domain evidence: Brunei Darussalam—Time-to-surgery target or reporting, osteoporosis assessment, anti-osteoporosis medication initiation, funding, governance, or sustainability.	Country-level policy and service-landscape evidence.	Grey literature
11	Fragility Fracture Network [13]. Asia Pacific Region > Brunei D. (2022). Accessed: May 18, 2026: https://fragilityfracturenetwork.org/asia-pacific-region-brunei-d/	Brunei Darussalam	Country/institutional context	Network webpage	Final country-domain evidence: Brunei Darussalam—National guideline or pathway.	Institutional implementation evidence.	Grey literature
12	Teo SP [14]. Audit of hip fractures and standards of care in Brunei Darussalam. J Med Biosci. 2022, 4:19-24. 10.14456/jmbs.2022.3. Available from: https://doi.org/10.14456/jmbs.2022.3.	Brunei Darussalam	Country/institutional context	Original journal article	Final country-domain evidence: Brunei Darussalam—Registry or audit system, orthogeriatric co-management, falls assessment or prevention, fracture liaison service, functional or discharge outcome reporting.	Institutional implementation evidence.	Grey literature
13	International Osteoporosis Foundation [15]. Indonesia country report. (2025). Accessed: May 19, 2026: https://www.osteoporosis.foundation/sites/iofbonehealth/files/2025-11/Indonesia_Final.pdf	Indonesia	Country level	Country report	Final country-domain evidence: Indonesia—National guideline or pathway, time-to-surgery target or reporting, falls assessment or prevention, osteoporosis assessment, anti-osteoporosis medication initiation.	Country-level policy and service-landscape evidence.	Grey literature
14	Fragility Fracture Network [16]. Fragility Fracture Network Indonesia. (2025). Accessed: May 18, 2026: https://fragilityfracturenetwork.org/fragility-fracture-network-indonesia/	Indonesia	Country/institutional context	Network webpage	Final country-domain evidence: Indonesia—Early mobilisation or rehabilitation.	Institutional implementation evidence.	Grey literature
15	Isnoni et al. [17]: Pre-injury demographic patterns of patients sustaining hip fractures in Malaysia. Malays Orthop J. 2012, 6:11-5. 10.5704/moj.1211.004. Available from: https://doi.org/10.5704/moj.1211.004.	Malaysia	Country/institutional context	Journal article	Corroborates the registry/audit domain through structured hip-fracture case-mix data capture.	Institutional implementation evidence.	Database
16	Cortez KA, Lai JGL, Tabu IA [29]. Economic burden and the effects of early versus delayed hospitalization on the treatment cost of patients with acute fragility hip fractures under the UPM-PGH orthogeriatric multidisciplinary fracture management model and fracture liaison service. Osteoporos Sarcopenia. 2021, 7:63-68. 10.1016/j.afos.2021.05.004. Available from: https://doi.org/10.1016/j.afos.2021.05.004.	Philippines	Regional	Journal article	Corroborates orthogeriatric co-management and fracture-liaison-service domains.	Regional implementation evidence.	Database
17	International Osteoporosis Foundation [18]. Malaysia country report. (2025). Accessed: May 19, 2026: https://www.osteoporosis.foundation/sites/iofbonehealth/files/2025-11/Malaysia_Final.pdf	Malaysia	Country level	Country report	Corroborates osteoporosis-assessment, medication-initiation, fracture-liaison-service and governance/sustainability domains.	Country-level policy and service-landscape evidence.	Grey literature
18	Lim WX, Khor HM, Lee JK, Ong T [19]. Fracture liaison service and its role in secondary fracture prevention in Malaysia: a scoping review. Malays Orthop J. 2023, 17:1-6. 10.5704/MOJ.2307.001. Available from: https://doi.org/10.5704/MOJ.2307.001.	Malaysia	Country/institutional context	Scoping review	Final country-domain evidence: Malaysia—National guideline or pathway, funding, governance, or sustainability.	Institutional implementation evidence.	Database
19	Klappenbach R, Lartigue B, Beauchamp M, Boietti B, Santero M, Bosque L, Monteverde E [20]. Hip fracture registries in low- and middle-income countries: a scoping review. Arch Osteoporos. 2023, 18:51. 10.1007/s11657-023-01241-x. Available from: https://doi.org/10.1007/s11657-023-01241-x.	Regional/multicountry (LMICs)	Country/institutional context	Scoping review	Final country-domain evidence: Malaysia—Registry or audit system, time-to-surgery target or reporting.	Institutional implementation evidence.	Database
20	Ong T, Khor HM, Kumar CS, Singh S, Chong E, Ganthel K, Lee JK [21]. The current and future challenges of hip fracture management in Malaysia. Malays Orthop J. 2020, 14:16-21. 10.5704/MOJ.2011.004. Available from: https://doi.org/10.5704/MOJ.2011.004.	Malaysia	Country/institutional context	Journal article	Final country-domain evidence: Malaysia—Mortality reporting.	Institutional implementation evidence.	Database
21	Yeap et al. [22]: Trends in post osteoporotic hip fracture care from 2010 to 2014 in a private hospital in Malaysia. Osteoporos Sarcopenia. 2017, 3:112-116. 10.1016/j.afos.2017.05.001. Available from: https://doi.org/10.1016/j.afos.2017.05.001.	Malaysia	Institutional	Journal article	Final country-domain evidence: Malaysia—Osteoporosis assessment, anti-osteoporosis medication initiation.	Institutional service-level evidence.	Database
22	Ministry of Health Malaysia [23]. Model of care: Management of Hip fracture Hospital Kuala Lumpur. (2024). Accessed: May 18, 2026: https://moh-idepository.nih.gov.my/uploadbahan/MODEL_OF_CARE_MANAGEMENT_OF_HIP_FRACTURE_HOSPITAL_KUALA_LUMPUR_2024.pdf	Malaysia	Institutional	Government model-of-care document	Final country-domain evidence: Malaysia—Orthogeriatric co-management.	Institutional service-level evidence.	Grey literature
23	Fragility Fracture Network [24]. Asia Pacific Region: Malaysia. (2022). Accessed: May 18, 2026: https://fragilityfracturenetwork.org/asia-pacific-region-malaysia/	Malaysia	Country/institutional context	Network webpage	Final country-domain evidence: Malaysia—Funding, governance, or sustainability.	Institutional implementation evidence.	Grey literature
24	International Osteoporosis Foundation [25]. Malaysia CTF final country check: 28 listed FLS services on Capture the Fracture Map of Best Practice. (2026). Accessed: May 19, 2026: https://www.capturethefracture.org/map-of-best-practice	Malaysia	Country/institutional context	Service-directory country extract	Final country-domain evidence: Malaysia—Fracture liaison service.	Institutional implementation evidence.	Grey literature
25	Myanmar Society of Endocrinology and Metabolism [26]. Myanmar Clinical Practice Guidelines for Osteoporosis. (2012). Accessed: May 18, 2026: https://msem.org.mm/wp-content/uploads/2023/10/Myanmar-Clinical-Practice-Guideline-for-Osteoporosis.pdf	Myanmar	Country/institutional context	Clinical guideline	Final country-domain evidence: Myanmar—National guideline or pathway, early mobilisation or rehabilitation, falls assessment or prevention, anti-osteoporosis medication initiation.	Institutional implementation evidence.	Grey literature
26	Fragility Fracture Network [27]. Asia Pacific Region: Myanmar. (2022). Accessed: May 18, 2026: https://fragilityfracturenetwork.org/asia-pacific-region-myanmar/	Myanmar	Country/institutional context	Network webpage	Final country-domain evidence: Myanmar—Registry or audit system, Osteoporosis assessment.	Institutional implementation evidence.	Grey literature
27	International Osteoporosis Foundation [28]. Myanmar country report. (2025). Accessed: May 19, 2026: https://www.osteoporosis.foundation/sites/iofbonehealth/files/2025-11/Myanmar_Final.pdf	Myanmar	Country level	Country report	Final country-domain evidence: Myanmar—National guideline or pathway, anti-osteoporosis medication initiation, fracture liaison service, funding, governance, or sustainability.	Country-level policy and service-landscape evidence.	Grey literature
28	Fragility Fracture Network Philippines [30]. Framework for a Sustainable Digital Health Registry. (2022). Accessed: May 18, 2026: https://www.ffnphil.org/framework-for-a-sustainable-digital-health-registry	Philippines	Country/institutional context	Framework/webpage	Corroborates registry/audit and governance/sustainability domains.	Institutional implementation evidence.	Grey literature
29	Fragility Fracture Network Philippines [31]. Projects: Multicenter Implementation of OrthoGeriatrics and FLS for Elderly Hip Fractures. (2023). Accessed: May 18, 2026: https://www.ffnphil.org/projects	Philippines	Regional	Institutional project webpage	Corroborates orthogeriatric co-management, fracture-liaison-service and governance/sustainability domains.	Regional implementation evidence.	Grey literature
30	Fragility Fracture Network [32]. Asia Pacific Region: Philippines. (2022). Accessed: May 18, 2026: https://fragilityfracturenetwork.org/asia-pacific-region-philippines/	Philippines	Country/institutional context	Network webpage	Corroborates registry/audit, fracture-liaison-service and governance/sustainability domains.	Institutional implementation evidence.	Grey literature
31	International Osteoporosis Foundation [33]. Philippines CTF final country check: 18 listed FLS services on Capture the Fracture Map of Best Practice. (2026). Accessed: May 19, 2026: https://www.capturethefracture.org/map-of-best-practice	Philippines	Country/institutional context	Service-directory country extract	Corroborates the fracture-liaison-service domain.	Institutional implementation evidence.	Grey literature
32	International Osteoporosis Foundation [34]. Philippines country report. (2025). Accessed: May 19, 2026: https://www.osteoporosis.foundation/sites/iofbonehealth/files/2025-11/Philippines_Final.pdf	Philippines	Country level	Country report	Final country-domain evidence: Philippines—Registry or audit system, national guideline or pathway, time-to-surgery target or reporting, fracture liaison service.	Country-level policy and service-landscape evidence.	Grey literature
33	Philippine Hip and Knee Society; Philippine Orthopaedic Trauma Society [35]. Guidelines on surgery for fragility hip fractures during the COVID-19 pandemic. (2020). Accessed: May 18, 2026: https://pcs.org.ph/wp-content/uploads/2022/06/POA_PHKS-and-POTS_Final-Guidelines-on-Surgery-for-Fragility-Hip-Fractures-During-the-Covid-19-Pandemic.pdf	Philippines	Country/institutional context	Clinical guideline	Final country-domain evidence: Philippines—National guideline or pathway.	Institutional implementation evidence.	Grey literature
34	Peña MARG, Tabu IA, Dy Ching Bing-Agsaoay D [36]. Functional outcomes among geriatric fragility hip fracture patients in a developing country: a comparative study between complete and incomplete post-operative rehabilitation. Acta Med Philipp. 2022, 56:133-142. 10.47895/amp.v56i6.3436. Available from: https://doi.org/10.47895/amp.v56i6.3436.	Philippines	Country/institutional context	Original journal article	Final country-domain evidence: Philippines—Orthogeriatric co-management, early mobilisation or rehabilitation, osteoporosis assessment, fracture liaison service, functional or discharge outcome reporting, funding, governance, or sustainability.	Institutional implementation evidence.	Grey literature
35	Ng JPH, Ho SWL, Yam MGJ, Tan TL [37]. Functional outcomes of patients with schizophrenia after hip fracture surgery: a 1-year follow-up from an institutional hip fracture registry. J Bone Joint Surg Am. 2021, 103:786-794. 10.2106/jbjs.20.01652. Available from: https://doi.org/10.2106/jbjs.20.01652.	Singapore	Institutional	Journal article	Final country-domain evidence: Singapore—Functional or discharge outcome reporting.	Institutional service-level evidence.	Database
36	Daruwalla ZJ, Wong KL, Pillay KR, Leong KM, Murphy DP [38]. Does ageing Singapore need an electronic database of hip fracture patients? The value and role of a national joint registry and an electronic database of intertrochanteric and femoral neck fractures. Singapore Med J. 2014, 55:287-8. 10.11622/smedj.2014072. Available from: https://doi.org/10.11622/smedj.2014072.	Singapore	National	Letter to the Editor	Corroborates the registry/audit domain.	National policy and implementation evidence.	Database
37	Koh GC, Tai BC, Ang LW, Heng D, Yuan JM, Koh WP [39]. All-cause and cause-specific mortality after hip fracture among Chinese women and men: the Singapore Chinese health study. Osteoporos Int. 2013, 24:1981-9. 10.1007/s00198-012-2183-7. Available from: https://doi.org/10.1007/s00198-012-2183-7.	Singapore	Country/institutional context	Journal article	Final country-domain evidence: Singapore—Mortality reporting.	Institutional implementation evidence.	Database
38	International Osteoporosis Foundation [40]. Singapore CTF final country check: 3 listed FLS services on Capture the Fracture Map of Best Practice. (2026). Accessed: May 19, 2026: https://www.capturethefracture.org/map-of-best-practice	Singapore	Country/institutional context	Service-directory country extract	Corroborates the fracture liaison service domain.	Institutional implementation evidence.	Grey literature
39	Yong et al. [41]: Risk factors and trends associated with mortality among adults with hip fracture in Singapore. JAMA Netw Open. 2020, 3:e1919706. 10.1001/jamanetworkopen.2019.19706. Available from: https://doi.org/10.1001/jamanetworkopen.2019.19706.	Singapore	Country/institutional context	Journal article	Final country-domain evidence: Singapore—Registry or audit system, mortality reporting.	Institutional implementation evidence.	Database
40	Lim et al. [42]: The role of prefracture health status in physical and mental function after hip fracture surgery. J Am Med Dir Assoc. 2018, 19:989-994.e2. 10.1016/j.jamda.2018.05.018. Available from: https://doi.org/10.1016/j.jamda.2018.05.018.	Singapore	Country/institutional context	Journal article	Final country-domain evidence: Singapore—Functional or discharge outcome reporting.	Institutional implementation evidence.	Database
41	Mok WQ, Jagadish UM, Yiap PL, Yu LH, Lim SM, Ker SY [43]. Promoting smoother and faster recovery for elderly sustaining hip fractures - a dedicated integrated care unit. Int J Integr Care. 2017, 17:431. 10.5334/ijic.3751. Available from: https://doi.org/10.5334/ijic.3751.	Singapore	Country/institutional context	Conference abstract	Final country-domain evidence: Singapore—Orthogeriatric co-management.	Institutional implementation evidence.	Database
42	Khoo Teck Puat Hospital [44]. Hip Fracture Service. (2026). Accessed: May 18, 2026: https://www.ktph.com.sg/service/medicalspecialties/Pages/orthopaedic.aspx	Singapore	Country/institutional context	Institutional service webpage	Final country-domain evidence: Singapore—Orthogeriatric co-management, early mobilisation or rehabilitation, falls assessment or prevention.	Institutional implementation evidence.	Grey literature
43	Chandran M, Mitchell PJ [45]. Tackling osteoporosis and fragility fractures in Singapore. Ann Acad Med Singap. 2021, 50:291-293. 10.47102/annals-acadmedsg.2021119. Available from: https://doi.org/10.47102/annals-acadmedsg.2021119.	Singapore	Country/institutional context	Journal editorial	Final country-domain evidence: Singapore—Osteoporosis assessment, anti-osteoporosis medication initiation, fracture liaison service, funding, governance, or sustainability.	Institutional implementation evidence.	Grey literature
44	International Osteoporosis Foundation [46]. Singapore country report. (2025). Accessed: May 19, 2026: https://www.osteoporosis.foundation/sites/iofbonehealth/files/2025-11/Singapore_Final.pdf	Singapore	Country level	Country report	Final country-domain evidence: Singapore—Registry or audit system, national guideline or pathway, time-to-surgery target or reporting, fracture liaison service, funding, governance, or sustainability.	Country-level policy and service-landscape evidence.	Grey literature
45	Amphansap T, Stitkitti N, Dumrongwanich P [47]. Evaluation of police general hospital's fracture liaison service (PGH's FLS): the first study of a fracture liaison service in Thailand. Osteoporos Sarcopenia. 2016, 2:238-243. 10.1016/j.afos.2016.09.002. Available from: https://doi.org/10.1016/j.afos.2016.09.002.	Thailand	Institutional	Journal article	Corroborates fracture-liaison-service, osteoporosis-assessment and medication-initiation domains.	Institutional service-level evidence.	Database
46	International Osteoporosis Foundation [48]. Thailand CTF final country check: 23 listed FLS services on Capture the Fracture Map of Best Practice. (2026). Accessed: May 19, 2026: https://www.capturethefracture.org/map-of-best-practice	Thailand	Country/institutional context	Service-directory country extract	Corroborates the fracture-liaison-service domain.	Institutional implementation evidence.	Grey literature
47	Charoenngam N, Pongchaiyakul C [49]. Current issues in evaluation and management of osteoporosis in Thailand. Osteoporos Sarcopenia. 2023, 9:53-59. 10.1016/j.afos.2023.05.002. Available from: https://doi.org/10.1016/j.afos.2023.05.002.	Thailand	Country/institutional context	Journal article	Final country-domain evidence: Thailand—National guideline or pathway.	Institutional implementation evidence.	Database
48	Stitkitti N, Amphansap T, Therdyothin A [50]. Mortality and outcome in fragility hip fracture care during COVID-19 pandemic in police general hospital, Thailand. Osteoporos Sarcopenia. 2023, 9:22-26. 10.1016/j.afos.2023.03.001. Available from: https://doi.org/10.1016/j.afos.2023.03.001.	Thailand	Institutional	Journal article	Final country-domain evidence: Thailand—Mortality reporting.	Institutional service-level evidence.	Database
49	Chotiyarnwong et al. [51]: Three-year outcomes of a fracture liaison service model at a university-based tertiary care hospital in Thailand. Arch Osteoporos. 2023, 18:26. 10.1007/s11657-023-01215-z. Available from: https://doi.org/10.1007/s11657-023-01215-z.	Thailand	Country/institutional context	Journal article	Final country-domain evidence: Thailand—Falls assessment or prevention, osteoporosis assessment, anti-osteoporosis medication initiation, fracture liaison service, functional or discharge outcome reporting.	Institutional implementation evidence.	Database
50	Anusitviwat C, Vanitcharoenkul E, Chotiyarnwong P, Unnanuntana A [52]. Surgical treatment for fragility hip fractures during the COVID-19 pandemic resulted in lower short-term postoperative functional outcome and a higher complication rate compared to the pre-pandemic period. Osteoporos Int. 2022, 33:2217-2226. 10.1007/s00198-022-06485-w. Available from: https://doi.org/10.1007/s00198-022-06485-w.	Thailand	Country/institutional context	Journal article	Corroborates the functional/discharge outcome-reporting domain.	Institutional implementation evidence.	Database
51	Sura-Amonrattana U, Tharmviboonsri T, Unnanuntana A, Tantigate D, Srinonprasert V [53]. Evaluation of the implementation of multidisciplinary fast-track program for acute geriatric hip fractures at a university hospital in resource-limited settings. BMC Geriatr. 2021, 21:548. 10.1186/s12877-021-02509-y. Available from: https://doi.org/10.1186/s12877-021-02509-y.	Thailand	Institutional	Journal article	Final country-domain evidence: Thailand—Orthogeriatric co-management, delirium or cognitive assessment, early mobilisation or rehabilitation, mortality reporting, functional or discharge outcome reporting.	Institutional service-level evidence.	Database
52	Songpatanasilp et al. [54]: Thai osteoporosis foundation (TOPF) position statements on management of osteoporosis. Osteoporos Sarcopenia. 2016, 2:191-207. 10.1016/j.afos.2016.10.002. Available from: https://doi.org/10.1016/j.afos.2016.10.002.	Thailand	Country/institutional context	Journal article	Final country-domain evidence: Thailand—National guideline or pathway.	Institutional implementation evidence.	Database
53	Sucharitpongpan W, Khampang R, Leelahavarong P, Valleenukul T, Srinonprasert V [55]. Integrated falls and hip fractures prevention in Thai older adults (Thai-IFHP), the falls surveillance system to prevent hip fractures and hip fracture care to prevent re-fracture: a study protocol of a pragmatic clinical trial [PREPRINT]. SSRN. 2026. 10.2139/ssrn.6134914. Available from: https://doi.org/10.2139/ssrn.6134914.	Thailand	Country/institutional context	Study protocol/preprint	Final country-domain evidence: Thailand—Registry or audit system.	Institutional implementation evidence.	Database
54	International Osteoporosis Foundation [56]. Thailand country extract from The Asia Pacific Regional Audit: Epidemiology, costs and burden of osteoporosis in 2025. (2025). Accessed: May 19, 2026: https://www.osteoporosis.foundation/sites/iofbonehealth/files/2025-11/Thailand_Final.pdf	Thailand	Country level	Country report/extract	Final country-domain evidence: Thailand—Time-to-surgery target or reporting.	Country-level policy and service-landscape evidence.	Grey literature
55	International Osteoporosis Foundation [57]. Viet Nam CTF final country check: 5 listed FLS services on Capture the Fracture Map of Best Practice. (2026). Accessed: May 19, 2026: https://www.capturethefracture.org/map-of-best-practice	Viet Nam	Country/institutional context	Service-directory country extract	Corroborates the fracture-liaison-service domain.	Institutional implementation evidence.	Grey literature
56	International Osteoporosis Foundation [58]. Vietnam country extract from The Asia Pacific Regional Audit: Epidemiology, costs and burden of osteoporosis in 2025. (2025). Accessed: May 19, 2026: https://www.osteoporosis.foundation/sites/iofbonehealth/files/2025-11/Vietnam_Final.pdf	Viet Nam	Country level	Country report/extract	Final country-domain evidence: Viet Nam—National guideline or pathway, time-to-surgery target or reporting, osteoporosis assessment, anti-osteoporosis medication initiation, fracture liaison service, funding, governance, or sustainability.	Country-level policy and service-landscape evidence.	Grey literature
57	Siow WS, Tay L, Mah CL [59]. Quality improvement initiative: how the setting up of an anaesthesia consultant-led perioperative outreach service addressed anaesthesia-specific issues to improve anaesthesia consult and surgery timings for hip fracture patients. BMJ Open Qual. 2022, 11:e001738. 10.1136/bmjoq-2021-001738. Available from: https://doi.org/10.1136/bmjoq-2021-001738.	Singapore	Country/institutional context	Journal article	Corroborates the time-to-surgery reporting domain.	Institutional implementation evidence.	Database
58	Tabu I, Ivers R, Costa ML [60]. Looking after patients with hip fracture in low- and middle-income countries. Bone Joint J. 2024, 106-B:1369-1371. 10.1302/0301-620x.106b12.bjj-2024-0652. Available from: https://doi.org/10.1302/0301-620x.106b12.bjj-2024-0652.	Regional/multicountry (LMICs)	Regional	Journal article	Corroborates registry/audit, guideline/pathway, orthogeriatric, rehabilitation, falls-prevention, osteoporosis-assessment and medication-initiation domains.	Regional implementation evidence.	Database
59	Fragility Fracture Network [61]. Hip Fracture Audit. (2026). Accessed: May 19, 2026: https://fragilityfracturenetwork.org/hip-fracture-audit/	International/toolbox	Regional	Network implementation webpage	Contributes a registry/audit, public benchmarking and governance/sustainability implementation framework.	Regional implementation evidence.	Grey literature

Supplementary methods: Search strategy

PubMed (NLM)

Search executed: 19 May 2026. Fields: All Fields.

(("hip fracture" OR "fragility fracture" OR "osteoporotic fracture")

AND

(registry OR audit OR "quality indicator" OR "performance indicator" OR pathway OR guideline OR orthogeriatric OR

"fracture liaison service" OR "secondary fracture prevention")

AND

(Singapore OR Malaysia OR Thailand OR Indonesia OR Philippines OR Vietnam OR "Viet Nam" OR Cambodia OR Laos OR "Lao PDR" OR

Myanmar OR Brunei OR ASEAN OR "Southeast Asia"))

Records retrieved: 149.

Search-term note: PubMed/MEDLINE used the displayed text-word strategy in All Fields; no MeSH field restriction was applied.

Embase (Elsevier)

Search executed: 19 May 2026.

('hip fracture'/exp OR 'hip fracture':ti,ab,kw OR 'fragility fracture':ti,ab,kw OR 'osteoporotic fracture':ti,ab,kw)

AND

('registry'/exp OR registry:ti,ab,kw OR audit:ti,ab,kw OR 'quality indicator'/exp OR 'quality indicator':ti,ab,kw OR

'performance indicator':ti,ab,kw OR pathway:ti,ab,kw OR guideline:ti,ab,kw OR orthogeriatric:ti,ab,kw OR

'fracture liaison service':ti,ab,kw OR 'secondary fracture prevention':ti,ab,kw)

AND

(singapore:ti,ab,kw OR malaysia:ti,ab,kw OR thailand:ti,ab,kw OR indonesia:ti,ab,kw OR philippines:ti,ab,kw OR

vietnam:ti,ab,kw OR 'viet nam':ti,ab,kw OR cambodia:ti,ab,kw OR laos:ti,ab,kw OR 'lao pdr':ti,ab,kw OR

myanmar:ti,ab,kw OR brunei:ti,ab,kw OR asean:ti,ab,kw OR 'southeast asia':ti,ab,kw)

Records retrieved: 413.

Search-term note: Embase used exploded Emtree terms for hip fracture, registry, and quality indicator, supplemented by title/abstract/keyword text words as displayed.

CINAHL Complete (EBSCOhost)

Search executed: 19 May 2026.

((MH "Hip Fractures+") OR TI ("hip fracture" OR "fragility fracture" OR "osteoporotic fracture") OR

AB ("hip fracture" OR "fragility fracture" OR "osteoporotic fracture"))

AND

((MH "Registries+") OR TI (registry OR audit OR "quality indicator" OR "performance indicator" OR pathway OR guideline OR

orthogeriatric OR "fracture liaison service" OR "secondary fracture prevention") OR AB (registry OR audit OR

"quality indicator" OR "performance indicator" OR pathway OR guideline OR orthogeriatric OR "fracture liaison service" OR

"secondary fracture prevention"))

AND

(TI (Singapore OR Malaysia OR Thailand OR Indonesia OR Philippines OR Vietnam OR "Viet Nam" OR Cambodia OR Laos OR "Lao PDR" OR

Myanmar OR Brunei OR ASEAN OR "Southeast Asia") OR AB (Singapore OR Malaysia OR Thailand OR Indonesia OR Philippines OR Vietnam OR

"Viet Nam" OR Cambodia OR Laos OR "Lao PDR" OR Myanmar OR Brunei OR ASEAN OR "Southeast Asia"))

Records retrieved: 17.

Search-term note: CINAHL Complete used CINAHL Headings MH "Hip Fractures+" and MH "Registries+", supplemented by title and abstract text words as displayed.

Grey Literature Search

Search executed: 18-19 May 2026.

Countries: Brunei Darussalam, Cambodia, Indonesia, Laos, Malaysia, Myanmar, the Philippines, Singapore, Thailand and Vietnam.

Sources: DuckDuckGo HTML; Ministry of Health/government, registry/audit, professional society and hospital programme websites; International Osteoporosis Foundation; Fragility Fracture Network; Capture the Fracture; Asia Pacific Fragility Fracture Alliance.

<country> hip fracture registry

<country> hip fracture audit

<country> fragility fracture network

<country> fracture liaison service

<country> osteoporosis guideline hip fracture

<country> orthogeriatric hip fracture

<country> hip fracture guideline time to surgery

<country> national hip fracture database
